# Mechano-mechanical parametric coupling in MEMS between GHz and kHz frequency regimes at room temperature

**DOI:** 10.1038/s41378-025-01111-1

**Published:** 2026-01-09

**Authors:** MinHee Kwon, Holger Arthaber, Daniel Platz, Ulrich Schmid

**Affiliations:** 1 Institute of Sensor and Actuator Systems, TU Wien, Vienna, Austria; 2 Institute of Electrodynamics, Microwave and Circuit Engineering, TU Wien, Vienna, Austria

**Keywords:** Engineering, Electrical and electronic engineering

## Abstract

Microelectromechanical systems (MEMS) sensors have been widely used in various fields, but their performance is often limited by thermal fluctuations and detection noise. Inspired by advances in cavity optomechanics, which utilize parametric coupling for precision sensing and noise reduction, we explore a new approach to overcoming these limitations. We demonstrate a purely mechanical parametric coupling system that replaces the optical mode with a GHz surface acoustic wave (SAW) cavity. This system couples the GHz SAW cavity with a kHz micro-cantilever oscillator under ambient conditions, bridging vastly different frequency regimes within a unified framework. This mechano-mechanical coupling is experimentally demonstrated by the generation of red and blue sidebands in the frequency spectrum as direct evidence of energy exchange between the SAW cavity and multiple vibrational modes of the cantilever. Using the standard cavity optomechanics framework, we calculate the coupling strength *g*_0_, which is on the order of 10^−3^ Hz, and compare it with previously reported values in optomechanical and electromechanical systems. Our findings establish mechano-mechanical parametric coupling as a practical alternative to conventional optomechanical interactions, offering a new framework for integrating GHz and kHz mechanical resonators into silicon MEMS-compatible platforms.

## Introduction

Microelectromechanical systems (MEMS) sensors have been extensively studied and developed over the past several decades. They are applied in diverse fields, such as inertial sensors^1,2^, mass sensors^3^, and atomic force microscope (AFM) tips^4^. The compact size, low power consumption, and cost-effectiveness of MEMS sensors have made these devices indispensable elements of modern technology. However, despite their long development history and wide adoption in various fields, MEMS sensors still face fundamental noise limitations. These limitations are primarily driven by thermal fluctuations and detection noise, which constrain the precision and reliability of MEMS sensors, especially in high-performance applications^5–7^.

Recent advances in cavity optomechanics provide promising strategies for addressing the challenges of thermal fluctuations and detection noise^8–14^. The heart of cavity optomechanics lies in parametric coupling, which establishes an energy flow between high-frequency optical mode and low-frequency mechanical mode. This coupling enables simultaneous interactions between optical and mechanical modes, with applications in displacement sensing, magnetometry, atomic force microscopy, transduction and signal processing, and non-reciprocal transport of photons and phonons^15,16^. Inspired by these developments, we propose to adapt the principles of cavity optomechanics to overcome the inherent noise limitations in MEMS sensors. However, applying cavity optomechanical techniques in MEMS sensors brings significant challenges. Bulky optical components are typically required, making it difficult to integrate them into the compact designs of MEMS devices.

In this work, we introduce an approach that uniquely combines standard MEMS technology with the principles of cavity optomechanics to address these challenges by a device-integrated approach. By employing a mechanical resonator with GHz frequency, specifically a surface acoustic wave (SAW) resonator, instead of high-frequency optical cavities, we present a novel framework for enabling cavity-assisted readout and exploring potential strategies for noise suppression within a standard CMOS-compatible process. This approach takes the well-established advantages of GHz SAW resonators, such as miniaturization and compatibility with standard MEMS fabrication techniques^17^, while avoiding the complexities of aligning and stabilizing bulky optical components. Furthermore, all experiments in this study are conducted at room temperature and atmospheric pressure, reflecting real-world operating conditions for MEMS devices.

There exist cases where mode coupling a SAW cavity has been investigated within the optomechanical framework, specifically coupling between SAW modes and optical modes^18^. However, in these studies, the SAW mode does not replace the optical mode but rather couples to it, so this approach is fundamentally different from the purely mechanical systems we propose. Other previous works have explored purely mechanical mode coupling within a single resonator. A study by Mahboob et al.^19^ demonstrates that two mechanical modes in the hundreds of kHz range can be parametrically coupled, with the second mode acting as a phonon cavity. This study shows key insights into phonon cavity interactions without requiring an optical mode. Similarly, mechanical mode coupling has been demonstrated in twin-microbottle resonators operating within the MHz range^20^ and in micro-cantilevers within the tens of kHz to a few MHz range^21^. Although these studies show purely mechanical mode coupling, the interactions are confined within a single resonator without coupling to another component. In contrast, our work extends purely mechanical coupling by demonstrating a GHz-frequency mechanical mode that plays the role of a phonon cavity and couples to a lower-frequency kHz mechanical mode. Unlike previous approaches, our system utilizes a GHz SAW cavity as a high-frequency mode, like a phonon cavity, forming a coupled two-resonator system with a kHz-frequency micro-cantilever. Furthermore, operating in the GHz regime offers the advantage of a reduced thermal phonon occupation number, making it relevant for potential quantum applications. By bridging vastly different frequency regimes, this system enables parametric coupling in MEMS devices, where the GHz cavity mode modulates and exchanges energy with the kHz mechanical mode of the oscillator.

**Fig. 1:**
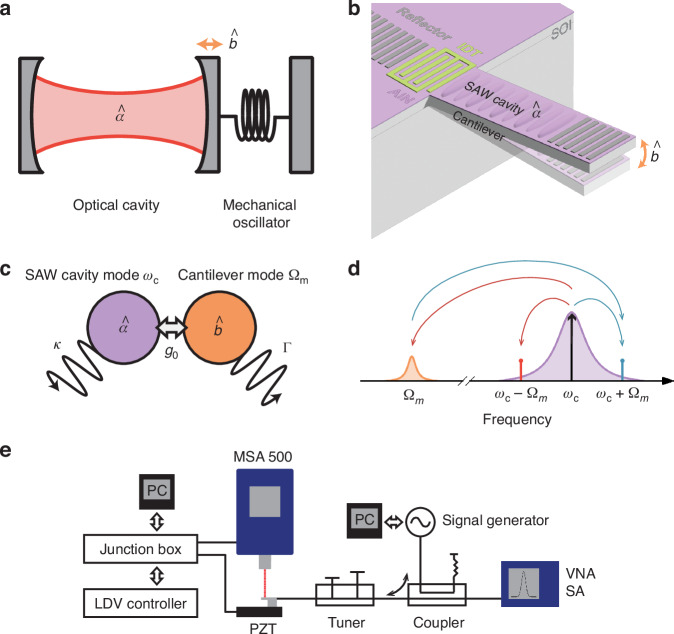
Conceptual illustration of mechano-mechanical parametric coupling inspired by cavity optomechanics and experimental setup. **a** Schematic of a conventional cavity optomechanical system. The system consists of an optical cavity mode $\widehat{a}$ and a mechanical oscillator mode $\widehat{b}$. **b** Schematic of the proposed system, where the SAW cavity mode replaces the optical cavity mode $\widehat{a}$, and the silicon micro-cantilever structure is the oscillator mode $\widehat{b}$. **c** Schematic of interaction between the SAW cavity mode $\widehat{a}$, with frequency *ω*_*c*_, and the cantilever mode $\widehat{b}$, with frequency Ω_*m*_. The interaction is characterized by the coupling strength *g*_0_, the mechanical damping rate Γ, and the cavity decay rate *κ*. **d** Conceptual frequency spectrum illustrating the SAW cavity mode at *ω*_*c*_, externally driven at the pump frequency (black arrow), and the resulting sidebands at *ω*_*c*_ ± Ω_*m*_ (red and blue lines) due to coupling with the mechanical mode Ω_*m*_. Red arrows represent energy emitted by the SAW cavity toward the oscillator, and blue arrows represent energy absorbed by the cavity from the oscillator. **e** Schematic of the experimental setup for measuring mechano-mechanical coupling. The laser Doppler vibrometer (LDV) system, including the MSA 500 and LDV controller, measures the mechanical displacement of the cantilever. A PZT actuator excites the cantilever, controlled through a junction box. The RF signal for exciting the SAW cavity mode is generated by a signal generator and passes through a coupler and tuner for impedance matching. The response of the SAW cavity is monitored using a Vector Network Analyzer (VNA), which can be switched to a Spectrum Analyzer (SA) mode to observe the sidebands in the frequency spectrum. A PC is used for data acquisition and control

To illustrate our system, Fig. 1 provides a conceptual overview of the architecture and framework of our proposed approach. Fig. 1a shows a conventional cavity optomechanical system, where an optical cavity mode $\widehat{a}$ with high frequency is confined by two mirrors and couples to the mechanical oscillator mode $\widehat{b}$ with low frequency. The interaction between these two modes can be described by the Hamiltonian^10,22^:

1 $$\begin{array}{ll}H=\hslash {\omega }_{c}{\widehat{a}}^{\dagger }\widehat{a}+\hslash {\Omega }_{m}{\widehat{b}}^{\dagger }\widehat{b}+{H}_{{\text{int}}},\\ {H}_{{\text{int}}}=-\hslash {g}_{0}{\widehat{a}}^{\dagger }\widehat{a}(\widehat{b}+{\widehat{b}}^{\dagger }).\end{array}$$

The first two terms in *H* represent the energies of the optical and mechanical modes, respectively. The interaction term, *H*_int_, describes the parametric coupling between two modes, with coupling strength *g*_0_. Fig. 1b introduces our proposed system, in which the optical cavity mode $\widehat{a}$ is replaced by a SAW resonator. Here, $\widehat{a}$ represents the annihilation operator for the SAW cavity mode with a frequency *ω*_*c*_ in the GHz range, while $\widehat{b}$ remains the annihilation operator for the mechanical oscillator mode, represented by a silicon micro-cantilever with a frequency *Ω*_*m*_ in the kHz range. The operators ${\widehat{a}}^{\dagger }$ and ${\widehat{b}}^{\dagger }$, which are the Hermitian adjoints of $\widehat{a}$ and $\widehat{b}$, correspond to the creation of excitations in the SAW and mechanical modes, respectively. Although the Hamiltonian formalism with creation and annihilation operators is a quantum description, in the classical mechanics regime, interpreting the operators as classical mode amplitudes, is an approach commonly used in cavity optomechanics. SAWs are generated by an interdigitated transducer (IDT) integrated on a piezoelectric aluminum nitride (AlN) film and propagate along the silicon micro-cantilever structure. Reflectors, acting as a mirror placed at the edge of the silicon micro-cantilever, confine the SAWs on the surface of the cantilever, forming the SAW cavity mode in a manner analogous to the optical cavity mode. Similar to how the motion of the mechanical oscillator affects the optical cavity mode in Fig. 1a, the vibration of the silicon micro-cantilever in Fig. 1b changes the length of the SAW cavity and changes the stress distribution within the SAW resonator. These changes affect the phase velocity of the SAWs, leading to a frequency shift in the SAW mode and establishing parametric coupling between the mechanical SAW cavity mode $\widehat{a}$ with high frequency (GHz) and the mechanical cantilever mode $\widehat{b}$ with low frequency (kHz). We refer to this interaction between purely mechanical modes over vast frequency intervals as mechano-mechanical coupling.

Figure 1c illustrates the interaction between the SAW cavity mode $\widehat{a}$, operating at a high frequency *ω*_*c*_, and the cantilever mode $\widehat{b}$, oscillating at a low frequency *Ω*_*m*_. The coupling strength *g*_0_ characterizes how the displacement of the cantilever parametrically leads to a frequency shift in the SAW mode. The cavity decay rate *κ* and the mechanical damping rate *Γ* determine the dissipation of energy from the system, affecting the strength of the interaction. As a result of the interaction, energy exchange between the SAW cavity and the cantilever mode manifests distinctly as sidebands in the frequency spectrum around the driven SAW resonance at *ω*_c_ ± Ω_*m*_, as shown in Fig. 1d. These sidebands result from the parametric modulation of the SAW resonance by the mechanically driven cantilever. In the experimental setup illustrated in Fig. 1e, the cantilever is excited at *Ω*_*m*_ via a piezoelectric shaker (with an applied PZT voltage in the range of 0–10 V), while a continuous RF tone at *ω*_*c*_ with fixed power (10 dBm) is applied to the SAW cavity by using a signal generator. Here, the excitation voltage always refers to the drive applied to the PZT actuator. This drive at *ω*_*c*_, indicated by the black arrow in Fig. 1d, pumps the SAW cavity. As a result of the interaction, energy exchange between the SAW cavity and the cantilever mode manifests distinctly as sidebands in the frequency spectrum around the driven SAW resonance at *ω*_c_ ± *Ω*_*m*_, as shown in Fig. 1d. Red arrows indicate energy emitted by the SAW cavity at the pump tone toward the oscillator, resulting in the red sideband. Blue arrows represent energy absorbed by the SAW cavity from the oscillator, giving rise to the blue sideband. Because the RF pump is applied on resonance with the SAW cavity (*ω*_*c*_), these emissive and absorptive processes occur at nearly equal rates, leading to approximately zero net energy flow. If either the mechanical excitation is applied at an off-resonance frequency or the RF tone is outside the SAW cavity mode, sidebands are not observed. The presence of sidebands around the driven SAW frequency at *ω*_*c*_ ± *Ω*_*m*_ provides direct evidence of parametric coupling between the two modes. Crucially, this sideband generation is fundamentally a parametrically nonlinear phenomenon. In a linear system, the output spectrum would only consist of frequency components already present in the input signal. In contrast, the parametric coupling term *H*_int_ of Eq. (1), involves the product of cavity and oscillator mode operators. Such interactions deviate from a linear input-output relationship, meaning that the system’s output frequencies differ from the input driving frequencies. This intrinsic nonlinearity underlies the observed generation of sidebands. Therefore, the primary focus of this study is to demonstrate these fundamental features of the mechano-mechanical coupling approach for future device applications that bridge GHz and kHz frequency regimes.

## Results

### SAW resonator and cantilever vibrational modes

**Fig. 2:**
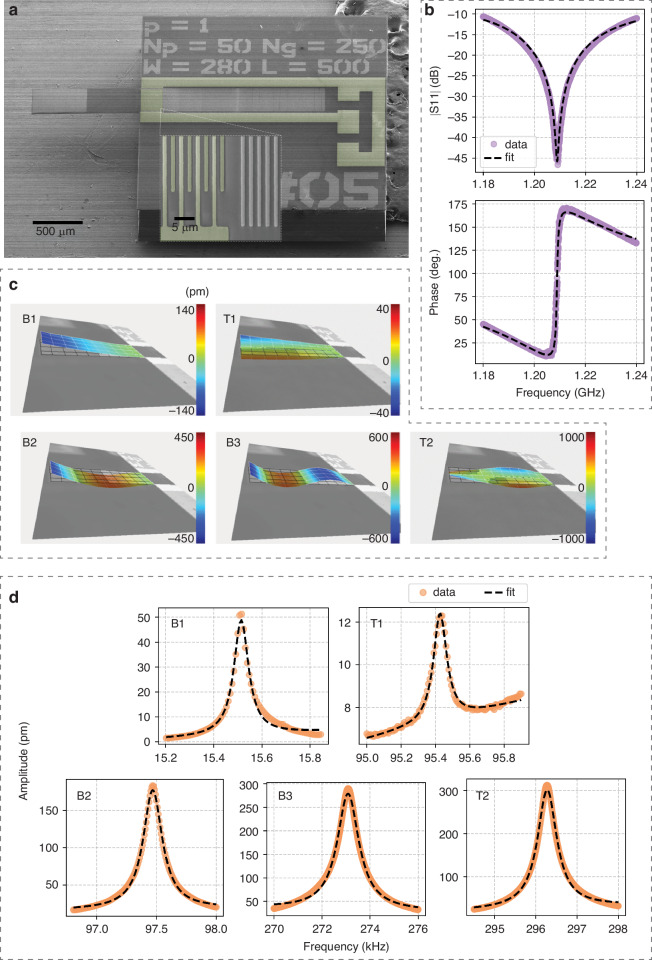
Characterization of the SAW resonator and the cantilever vibrational modes. **a** False-color SEM image of the device. Green regions indicate the IDT and electrode lines. **b** Reflection measurements of the SAW resonator near its resonance, showing the S11 magnitude and phase. Purple dots are data, and black dashed lines are fits. **c** LDV measurement showing spatial mode shapes of the cantilever for five observed modes: B1, T1, B2, B3, and T2. **d** Frequency response of the cantilever for each mode. The y-axis represents the displacement amplitude of the cantilever. Orange dots represent measurements, and black dashed lines show Lorentzian fits

Figure 2a shows a false-color scanning electron microscopy (SEM) image of the fabricated device, implementing the concept presented in Fig. 1b. The silicon micro-cantilever has a length of 1000 μm, a width of 308 μm, and a thickness of 11 μm. The green-colored region is the IDT with 50 electrode pairs and the busbars are extended to directly connect to the electrode pads. At the edge of the silicon micro-cantilever, a reflector is placed, consisting of 250 grating lines. On the opposite side of the IDT, another reflector is placed, consisting of over 500 grating lines. This second reflector is located directly next to the IDT without any cavity-free space on that side. This asymmetric design confines the SAW mode on the vibrating cantilever structure. The width of both the IDT electrodes and the grating structures is 1 μm, with a periodic spacing of 1 μm between finger pairs. The detailed fabrication process of the device is described in Section 4.1.

We first characterize the individual modes of both the SAW resonator and the cantilever. The resonance frequency of the SAW resonator is determined by reflection measurement using a vector network analyzer (VNA) under ambient conditions. Fig. 2b presents the measurement data of the SAW resonator, showing both the magnitude and phase of the reflection coefficient *S*_11_. The frequency response near resonance is analyzed by fitting the measured *S*_11_ data using a standard RLC circuit model:

2 $${S}_{11}(f)=\frac{({Q}_{e}-{Q}_{i})+2i{Q}_{i}{Q}_{e}\Delta f/{f}_{c}}{({Q}_{e}+{Q}_{i})+2i{Q}_{i}{Q}_{e}\Delta f/{f}_{c}},$$

where *f*_*c*_ is the resonance frequency, Δ*f* = *f* − *f*_*c*_ is the frequency detuning away from resonance, and *Q*_*i*_ and *Q*_*e*_ represent the internal and external quality factors (Q-factors), respectively^17,23^. From the fit, the resonance frequency *f*_*c*_ = *ω*_*c*_/2*π* is determined to be 1.21 GHz. To achieve critical matching conditions (*S*_11_ = 0 at *f*_*c*_), we used a microwave tuner to minimize signal reflection at the resonance frequency. This impedance matching is necessary to avoid too much power returning to the spectrum analyzer when driving the resonator by the source. The resonator is thus matched at *f*_*c*_ and is critically coupled, with *Q*_*e*_ ≈ *Q*_*i*_. From the fit, the internal quality factor is extracted as *Q*_*i*_ = 4328, characterizing the intrinsic loss of the SAW resonator.

The vibrational cantilever modes are characterized using a laser Doppler vibrometer (LDV) under ambient conditions. The resonance response of the cantilever is scanned over a frequency range up to 300 kHz, where a piezoelectric shaker excites the cantilever, revealing five distinct flexural vibration modes. The spatial mode shapes of the cantilever, including the first bending mode (B1), the first torsional mode (T1), the second bending mode (B2), the third bending mode (B3), and the second torsional mode (T2), are visualized in Fig. 2c. The frequency responses of these modes are presented in Fig. 2d, showing the measured displacement amplitudes of the cantilever. The resonance frequencies *f*_*m*_ = *Ω*_*m*_/2*π* are identified as 15.52 kHz, 95.45 kHz, 97.47 kHz, 273.08 kHz, and 296.23 kHz for B1, T1, B2, B3, and T2, respectively. The corresponding Q-factors are 212, 1027, 500, 250, and 526. The resonance frequencies and Q-factors of the cantilever modes are determined by fitting the measured frequency response with a standard Lorentzian function.

### Sidebands in SAW spectrum

**Fig. 3:**
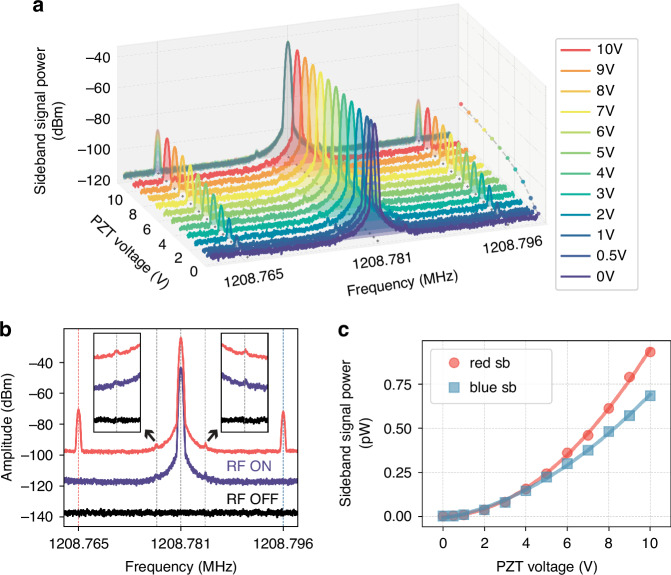
Observation of sidebands in the frequency spectrum. **a** Measured frequency spectrum around the SAW cavity frequency *ω*_*c*_/2*π* using a spectrum analyzer while sweeping the PZT voltage from 0 V to 10 V. The x-axis represents the frequency, the y-axis denotes the PZT voltage, and the z-axis corresponds to the signal power (dBm). A central peak at 1208.781 MHz results from the externally applied RF excitation at SAW cavity frequency *ω*_*c*_/2*π*. Red and blue sidebands appear symmetrically at (*ω*_*c*_ ± *Ω*_*B*1_)/2*π*, with increasing amplitudes as the PZT voltage increases. **b** Measured frequency spectrum with PZT actuation off (0 V) for RF pump ON (purple) and RF OFF (black). The red curve, which is vertically shifted by 20 dBm, corresponds to the data in Fig. 3a measured at 10 V. Small symmetric peaks appear at −3.66 kHz and +3.67 kHz near the central RF tone. Insets show zoomed views around the ±3.7 kHz offsets. **c** Extracted peak signal power (pW) of the red and blue sidebands as a function of PZT voltage. The red circles and blue squares represent the red sideband and the blue sideband, respectively. The solid lines indicate a quadratic fit

To investigate the interaction between the SAW cavity and the micro-cantilever, we measure the frequency spectrum around the SAW resonance with a spectrum analyzer while simultaneously driving the SAW cavity mode at its resonance frequency *ω*_*c*_/2*π* of 1.21 GHz by a signal generator and exciting the first bending mode (B1) of the micro-cantilever at its resonance frequency *Ω*_*B*1_/2*π* of 15.52 kHz using a PZT (Lead zirconate titanate) actuator (see Fig. 1e for the measurement setup). Figure 3a shows the SAW frequency spectrum around the SAW resonance frequency *ω*_*c*_/2*π* for different excitation voltages applied to the piezoelectric shaker ranging from 0 V to 10 V. A strong tone in the center at 1208.781 MHz, corresponding to the driven SAW resonance, with a signal power of approximately − 44 dBm. The signal generator generates a carrier tone with fixed amplitude at the SAW resonance frequency *ω*_*c*_, resulting in a constant central spectral peak that remains unchanged regardless of the applied PZT voltage. Although cantilever vibration can, in principle, change the transfer function of the SAW resonator, the externally applied carrier tone dominates the spectral response at *ω*_*c*_, so that the modulation induced by the cantilever motion appears primarily as sidebands rather than as measurable variations of the central peak. Red and blue sidebands are observed at frequencies offset by ±*Ω*_*B*1_/2*π* from *ω*_*c*_/2*π* at 1208.765 MHz and 1208.796 MHz, respectively. At the smallest excitation voltage of 0.5 V, the frequency differences between the SAW mode and the red and blue sidebands are 15.56 kHz symmetrically, which deviate slightly by 0.04 kHz from the excitation micro-cantilever frequency of 15.52 kHz. At the largest PZT actuator voltage of 10 V, the frequency differences are 15.59 kHz and 15.50 kHz for the red and blue sidebands, respectively, showing a slight asymmetry but remaining consistent within the resolution limit, considering both the frequency resolution of the spectrum analyzer and the resolution bandwidth used in our measurement, which is approximately 0.1 kHz. As the PZT voltage is increased, the amplitudes of both sidebands grow. This relationship is projected onto the PZT-voltage-signal-power plane, with the maximum signal power of the blue sideband indicated by circles.

In Fig. 3a, two very small responses consistently appear on either side of the central tone. Figure 3b shows frequency spectra measured with the PZT actuator turned off (no mechanical actuation and no sidebands) for both the RF pump ON (purple) and RF pump OFF (black). For comparison, the red spectrum represents one of the datasets shown in Fig. 3a, measured with the RF pump ON and the PZT driven at 10 V. To distinguish it from the other spectra, this red spectrum has been vertically shifted by 20 dBm. In this condition, where no mechanical excitation is applied to the cantilever, the sidebands from parametric coupling disappear, as expected. However, small symmetric peaks remain visible between the central tone and the sidebands, at frequency offsets of −3.66 kHz and +3.67 kHz from the RF carrier. These peaks are only present when the RF pump is active and disappear entirely when the RF is turned off. Furthermore, we observe the same symmetric peaks at around ±3.66 kHz from the RF pump frequency in measurements on other devices with different SAW cavity resonance frequencies and cantilever geometries (see Fig. S1 in Supplementary information). This consistent appearance of small peaks at the same offset, regardless of device-related parameters, indicates that they are not caused by mechano-mechanical parametric coupling or a physical response of the device. Instead, the fixed frequency offset and symmetric structure suggest that these responses originate from the RF instrumentation itself. A likely explanation is residual mixing in the signal generator, meaning that internal reference or modulation signals can leak into the output and produce small unwanted tones at fixed frequency offsets.

To quantify the observed increase in sideband amplitudes, the signal power is converted from dBm to pW, and the peak values of the red and blue sidebands are plotted for each PZT voltage, as shown in Fig. 3c. As the PZT voltage increases, the amplitudes of both sidebands grow, with the red sideband showing a larger amplitude increase than the blue sideband. The solid lines indicate a fit of a second-order polynomial function. This quadratic dependence on the PZT voltage is expected by frequency modulation caused by the mechanical stretching and compression of the resonator. The modulated signal can be expressed as $s(t)=A\cos [{\omega }_{c}t+\beta \sin ({\Omega }_{B1}t)]$, where the modulation index *β* is proportional to the cantilever displacement and thus to the applied PZT voltage. In the small-modulation limit, the first-order sideband amplitude follows the Bessel function *J*_1_(*β*) ≈ *β*/2, and the corresponding sideband power scales as ${J}_{1}^{2}(\beta )\propto {\beta }^{2}$. Although this modulation causes a shift in the SAW resonance frequency, such a shift is not observed in the VNA reflection spectrum, as any potential shift would be much smaller than the 3 dB bandwidth of the SAW cavity (360 kHz), as shown in Fig. 2b.

After having confirmed the coupling between the SAW cavity and B1 mode, we investigate whether similar coupling occurs in the higher-order mode of the cantilever. Like the process used for the B1 mode, we couple the SAW cavity mode with the higher-order vibrational modes of the micro-cantilever: T1, B2, B3, and T2. Each mode is excited at its respective resonance frequency, and the corresponding sidebands are also observed in the frequency spectrum. Similar to the coupling with the B1 cantilever mode, the coupling with higher-order cantilever modes (T1, B2, B3, and T2) generates red and blue sidebands. The amplitudes of sidebands grow asymmetrically as the PZT voltage increases (see Fig. S2 in Supplementary information).

**Fig. 4:**
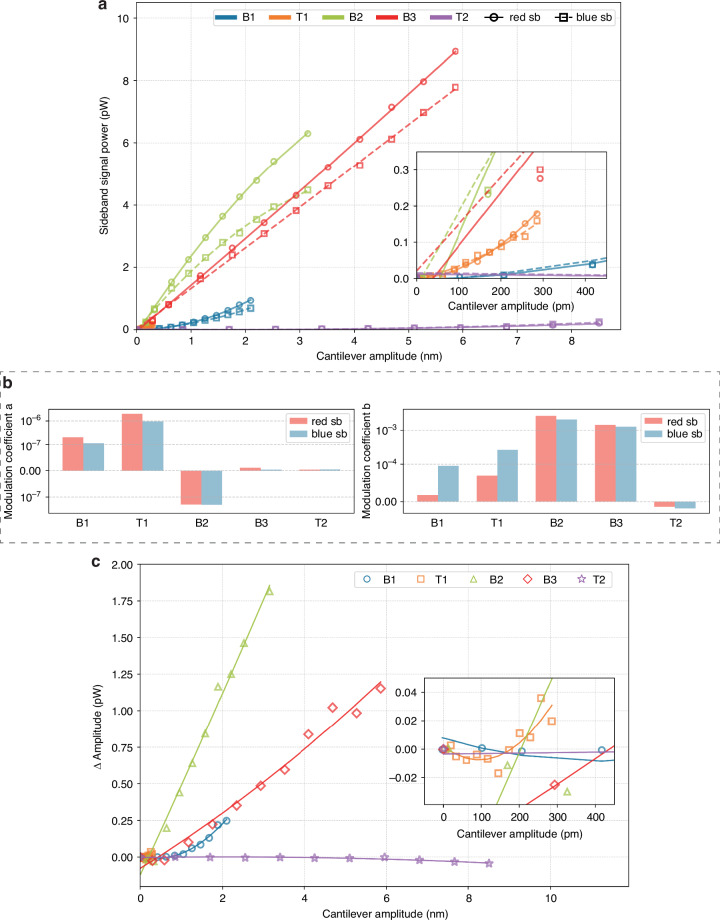
Analysis of sideband signal power as a function of cantilever amplitude between the SAW mode and the vibration modes (B1, T1, B2, B3, T2). **a** Sideband signal power for red (circles) and blue (squares) sidebands as a function of cantilever amplitude. The solid and dashed lines show a quadratic fit using Eq. (3). The inset shows the T1 mode, which is less visible in the main plot due to its lower signal level. **b** Bar plots the second-order modulation coefficient (*a*) and first-order modulation coefficient (*b*) extracted from the quadratic fit in **a**. **c** Asymmetry in sideband signal power, calculated as the difference between red and blue sideband amplitudes. The solid lines represent a quadratic fit. The inset provides a view of the T1 mode, which is less visible due to its lower signal level

However, the PZT voltage is an experimentally controlled parameter and does not equal the physical amplitude of the cantilever vibration. To quantitatively analyze how the cantilever motion affects the SAW cavity, we first need to determine the cantilever amplitude for a given PZT voltage. Therefore, the applied PZT voltage is converted using LDV measurements in the maximum displacement of the considered cantilever’s vibration mode to establish the relationship between the physical motion of the cantilever and the sideband signal in the SAW spectrum. We refer in this study to this maximum displacement as the cantilever amplitude for simplicity. The laser of the LDV is positioned on the cantilever at the location of maximum displacement for each mode, and the displacement is directly measured at PZT voltages of 1 V and 10 V. For modes B1, T1, and B2, the displacement is assumed to increase linearly between these PZT voltages. The maximum displacement location for each mode was identified from the mode scan results as shown in Fig. 2c. The LDV laser was subsequently fixed at the identified position of maximum displacement for each mode, and the displacement at the resonance frequency was measured ten times and averaged. The corresponding sideband power data for each mode were originally measured as a function of PZT voltage as shown in Fig. 3c and Fig. S2 (in Supplementary information). These PZT-dependent data are then converted to cantilever amplitude using the LDV measurements described above, enabling a unified representation in Fig. 4a, which directly relates physical displacement to sideband signal strength across all observed modes. Fig. 4a shows the relationship between the cantilever amplitude and the red and blue sideband power for all vibrational modes (B1, T1, B2, B3, and T2). The circles and squares represent the signal intensity of the red and blue sidebands, respectively. The sidebands generated by bending modes generally have higher signal power compared to torsional modes. The solid and dashed lines represent fits to the data based on the second-order polynomial equation:

3 $$S(d)=a\cdot {d}^{2}+b\cdot d+c,$$

where *S*(*d*) represents the sideband signal power (pW) as a function of the cantilever amplitude *d* (pm). Given the observed curvature in the sideband response, a second-order polynomial was chosen as a simple mathematical model that is not derived from an underlying physical theory. The coefficients *a* and *b*, obtained from the polynomial fit and are referred to as modulation coefficients. These terms characterize the curvature and slope of the data-driven fit between displacement and sideband power. The exact values of the coefficients *a* and *b* for each mode are provided in Fig. 4b. The second-order modulation coefficient *a* is largest for the T1 mode, reflecting the strongest curvature in the sideband amplitude as a function of cantilever amplitude, whereas it is smaller for the higher-order modes B3, and T2. In contrast, the B2 mode has a negative *a* value, indicating that the sideband amplitude increases but in a sublinear manner, with a reduced growth rate at higher cantilever amplitude. The first-order modulation coefficient *b* is comparatively larger for the B2 and B3 modes, with the smallest *b* observed for the T2 mode. This suggests that the B2 and B3 modes show a stronger linear increase in sideband amplitude at lower displacement, whereas T2 exhibits a weaker overall response.

Given that the red and blue sidebands generally show an asymmetric response, we investigate the amplitude asymmetry of sidebands as the cantilever amplitude increases. Fig. 4c presents the differences in signal power between the red and blue sidebands as a function of the cantilever amplitude, while Fig. 4a presents the total sideband power. The solid lines represent fits to the data based on a second-order polynomial. Across most modes, an increase in the cantilever amplitude leads to an increased power asymmetry between the red and blue sidebands. The B1, B2, and B3 modes show an increase in asymmetry, with the red sideband growing more significantly than the blue sideband. In contrast, the T2 mode shows symmetrical sidebands. The T1 mode shows that the blue sideband is initially larger at small displacements, but as the displacement increases, the red sideband is eventually larger as shown in the inset of Fig. 4c. These results indicate that, as the cantilever amplitude increases, bending modes (B1, B2, B3) generally exhibit stronger sideband asymmetry compared to torsional modes (T1, T2).

### Calculation of coupling strength *g*_0_

In this section, we aim to extract the coupling strength *g*_0_ in our mechano-mechanical system and to compare it with previous studies on cavity optomechanical systems. The coupling strength *g*_0_ is often extracted from an experimental shift in the cavity resonance frequency in cavity optomechanics. However, in our system, it is difficult to directly extract such a small shift due to the GHz cavity frequencies compared to the kHz mechanical modes, and the limited frequency resolution of our experimental setup. In particular, the expected resonance shift caused by the cantilever vibration is in the range of a few hertz up to a few hundred hertz, which is below our measurable resolution (17.5 kHz step). Therefore, we estimate *g*_0_ based on theoretical modeling combined with experimentally accessible parameters.

The parametric coupling in our system is governed by the Hamiltonian interaction *H*_int_ in Eq. (1), which describes how the mechanical mode induces a frequency shift in the SAW cavity mode. When the mode amplitudes are represented by classical variables, the coupled equations of motion are as

4 $$\dot{a}=\left(i\Delta-\frac{\kappa}{2}\right)a+i{g}_{0}a(b+{b}^{*})+E,$$

5 $$\dot{b}=\left(-i{\Omega }_{m}-\frac{{\Gamma }_{m}}{2}\right)b+i{g}_{0}| a|^{2},$$

where *E* represents the amplitude of the external drive applied to the cavity. The equations show how the displacement of the mechanical oscillator couples to the cavity resonance frequency with coupling strength *g*_0_. Within this standard optomechanical framework^10,22^, the coupling strength is defined as

6 $${g}_{0}={x}_{{\text{ZPF}}}\cdot G,$$

where *x*_ZPF_ is the zero-point fluctuation displacement of a mechanical mode within a classical system, and *G* represents the responsivity of the optical cavity’s angular resonance frequency *ω*_*c*_ to the effective change in the cavity length Δ*L*. In our system, the mechanical mode corresponds to the cantilever’s vibrational modes, and the SAW cavity mode replaces the optical cavity mode. The zero-point fluctuation displacement *x*_ZPF_ in our system is given by

7 $${x}_{{\text{ZPF}}}=\sqrt{\frac{\hslash }{2{m}_{{\rm{e}}\mathrm{ff}}{\Omega }_{m}}},$$

where *ℏ* is the reduced Planck constant, *m*_eff_ is the effective mass of the cantilever, and *Ω*_*m*_ is its angular resonance frequency. The values of *m*_eff_ for each cantilever mode are provided in Table 1, with detailed calculations described in Supplementary Information (Section S3).

**Table 1:** Parameters and calculated coupling strength *g*_0_ for the bending modes (B1, B2, B3)

Mode	*m*_eff_ (kg)	*Ω*_*m*_/2*π* (Hz)	Δ*ω*_*c*_/2*π* (Hz)	Δ*L* (m)	*g*_0_ (Hz)
B1	11.15 × 10^−10^	15.52 × 10^3^	3.79	1.58 × 10^−11^	10.47 × 10^−4^
B2	8.40 × 10^−10^	97.47 × 10^3^	38.28	8.96 × 10^−11^	8.59 × 10^−4^
B3	5.03 × 10^−10^	273.08 × 10^3^	183.28	25.16 × 10^−11^	11.32 × 10^−4^

In cavity optomechanics, the optical cavity resonance frequency shifts in response to changes in the cavity length, so the coupling coefficient is defined as

8 $$G=\frac{\partial {\omega }_{c}}{\partial L}\approx \frac{\Delta {\omega }_{c}}{\Delta L}.$$

Similarly, in our system, the SAW cavity mode is confined to the top surface of the cantilever, and its resonance frequency *ω*_*c*_ is affected by changes in the effective cavity length Δ*L*. In other words, as the cantilever bends during vibration, the length of the cantilever undergoes deformation, leading to a shift in the SAW resonance frequency Δ*ω*_*c*_. However, unlike optical cavities where length changes directly determine a shift in the cavity resonance frequency, in the SAW cavity, these length changes apply strain in the cantilever. Consequently, the SAW resonance frequency shift Δ*ω*_*c*_ originates not only from geometric cavity length changes but also from strain-induced changes in the material properties, such as density.

To obtain the SAW resonance frequency shift *Δω*_*c*_ when the cantilever is bent, we use the experimental relationship from GHz SAW strain sensors^24^ as

9 $$\Delta {\omega }_{c}=2\pi R\cdot {\epsilon }_{\max },$$

where *R* is the frequency shift of the SAW resonance *f*_*c*_ (*ω*_*c*_/2*π*) per strain *ε*, and ${\epsilon }_{\max }$ is the maximum strain on the top surface of the cantilever confining the SAWs. We adopt the responsivity *R* = 93.80 Hz/*μϵ* from a GHz SAW strain sensor^24^, which has similar material properties, resonance frequency, and resonator length to our SAW resonator. Since *R* represents the frequency shift per strain *ε*, the next step is to determine the actual applied strain due to the deformation of the cantilever surface. We calculate the maximum strain along the cantilever, which depends on its vibrational displacement. The applied strain can be derived from the cantilever’s mode shape function *w*(*x*), following the Euler–Bernoulli beam theory^25^. The mode shape function is given by

10 $$w(x)={w}_{C}\cdot \phi (x),$$

where *w*_*C*_ is the maximum displacement amplitude, and *ϕ*(*x*) is the normalized mode shape function satisfying

11 $$\mathop{\max }\limits_{x\in [0,L]}\phi (x)=1,$$

where *L* is the SAW cavity length. Since the strain on the topside of the cantilever is related to the curvature of the cantilever’s deformation, the maximum strain ${\epsilon }_{\max }$ can be expressed as

12 $${\epsilon }_{\max }=\mathop{\max }\limits_{x\in [0,L]}| -\frac{{t}_{{\text{cant}}}}{2}\cdot {w}_{C}\cdot \frac{{d}^{2}\phi (x)}{d{x}^{2}}| ,$$

where *t*_cant_ is the thickness of the cantilever. *ϕ*(*x*) is normalized, and its second derivative *d*^2^*ϕ*(*x*)/*dx*^2^ determines the curvature of the cantilever’s deformation.

To compare the coupling strength *g*_0_ in our system with *g*_0_ reported in previous studies, we keep the coupling coefficient *G* as the cavity mode frequency shift per unit change in effective cavity length Δ*L*, as given in Eq. (8). In our study, the resonance frequency shift Δ*ω*_*c*_ is determined using the experimental responsivity *R*, which relates to the maximum strain ${\epsilon }_{\max }$. To ensure consistency with Eq. (8), we compute the effective cavity length change Δ*L*, which accounts for the applied strain due to cantilever deformation. The SAW cavity length *L* is strictly different from the cantilever length because SAW cavity length includes the penetration depth in reflectors^17^. The SAW cavity length is approximately 300 μm longer than the cantilever length. However, since the change in the effective cavity length arises from the deformation of the cantilever, we approximate Δ*L* by integrating the strain distribution along the cantilever length *L*_cant_ as

13 $$\Delta L\approx {\int }_{0}^{{L}_{{\text{cant}}}}\epsilon (x)\,dx.$$

where *ϵ*(*x*) represents the strain distribution along the cantilever. The detailed derivation of Δ*L* is provided in the Supplementary Information. By substituting Eq. (9) and Eq. (13) into Eq. (8), we obtain the coupling coefficient *G* in our system. Next, we extract the coupling strength *g*_0_ by incorporating Eq. (7) and Eq. (8) into Eq. (6). The mode frequencies *Ω*_*m*_ and *ω*_*c*_ are directly measured from our experiments, while geometric parameters such as *L*_cant_ and *t*_cant_ are obtained from the fabricated device.

We calculate the coupling strength *g*_0_ for the interaction between the SAW cavity mode and the cantilever modes. We focus on calculating *g*_0_ for bending modes (B1, B2, B3), where the coupling effect is most significant. Table 1 summarizes the parameters and the resulting coupling strengths *g*_0_ for each bending mode. The calculated values of *g*_0_ range from 8.59 × 10^−4^ Hz to 11.32 × 10^−4^ Hz, reflecting variations in the effective mass, mode frequencies, and strain distribution.

### Second-order sidebands in mechano-mechanical system

To investigate whether our platform with SAW cavity and micro cantilever can generate higher-order sidebands beyond the first-order sidebands shown in Fig. 3a, we remeasured the spectrum with a wider frequency span to observe second-order sidebands. In cavity optomechanics, higher-order sidebands are regarded as a feature of nonlinear interactions, and second-order sidebands in particular have been theoretically linked to quantum phenomena such as photon blockade, effective Kerr nonlinearities, and the emergence of nonclassical states^26^. Experimental demonstrations, however, are typically challenging, as second-order sidebands usually require strong optical driving, cryogenic conditions, or additional probe tones in OMIT-type configurations^27–29^. In contrast, this study tests whether second-order sidebands can be detected at room temperature without additional probe tones, using only a single RF pump combined with mechanical actuation.

**Fig. 5:**
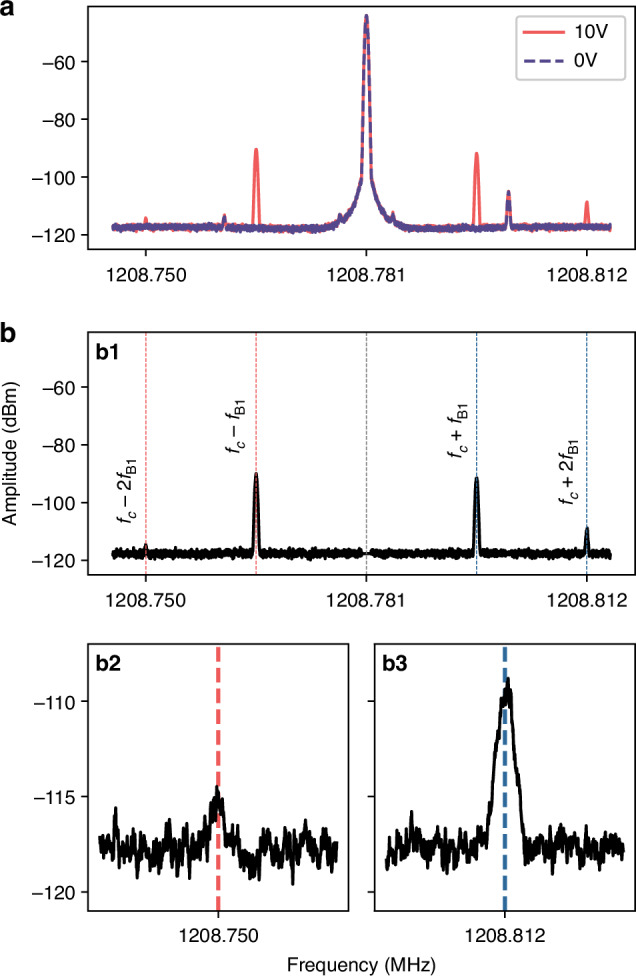
Observation of second-order sidebands in a mechano-mechanical system. **a** Measured frequency spectra, over a wider frequency range than in Fig. 3**a**, around the SAW cavity resonance frequency *f*_*c*_ (*ω*_*c*_/2*π*) with an RF pump tone applied at 1208.781 MHz and the cantilever driven at its first bending mode *f*_*B*1_ = 15.52 kHz (*Ω*_*B*1_/2*π*). Spectra are shown for PZT actuator voltages of 0 V (dashed purple) and 10 V (solid red). **b** Difference spectrum obtained by subtracting the 0 V spectrum from the 10 V spectrum in **a**, with vertical offset adjusted so that the amplitude of the red sideband at *f*_*c*_ − *f*_*B*1_ matches the original measurement. **b1** All observed sideband frequencies annotated, including both first- and second-order sidebands at *f*_*c*_ ± *f*_*B*1_ and *f*_*c*_ ± 2*f*_*B*1_. **b2** and **b3** Zoom-in views around *f*_*c*_ − 2*f*_*B*1_ and *f*_*c*_ + 2*f*_*B*1_, respectively, showing the second-order sidebands above the noise floor

As shown in Fig. 5a, in addition to the first-order sidebands at *f*_*c*_ ± *f*_*B*1_, smaller additional frequency components appear at *f*_*c*_ ± 2*f*_*B*1_. Fig. 5b displays the difference spectrum between the two measurements in Fig. 5a, with the amplitude offset adjusted so that the red sideband at *f*_*c*_ − *f*_*B*1_ matches its measured value in Fig. 5a. This subtraction removes the central RF pump carrier and other RF-related spurs, leaving only frequency components originating from the SAW cavity-cantilever mode coupling. As annotated in Fig. 5b1, both the first-order (*f*_*c*_ ± *f*_*B*1_) and second-order (*f*_*c*_ ± 2*f*_*B*1_) sidebands are identifiable. Because the second-order sidebands are much weaker, the zoomed-in views in Fig. 5b2, b3 are provided for clarity. The measured sideband frequencies agree with the expected positions, at *f*_*c*_ ± *f*_*B*1_ (1208.765 MHz and 1208.796 MHz) and *f*_*c*_ ± 2*f*_*B*1_ (1208.750 MHz and 1208.812 MHz). Using peak powers to define the ratio, the second-order sidebands correspond to approximately 0.42 % (red) and 2.07 % (blue) of the respective first-order sidebands.

## Discussion

While the concept of cavity optomechanics has been widely applied to electro-mechanical systems, our results first demonstrate that purely mechanical parametric coupling can be realized using a GHz SAW cavity and a kHz mechanical oscillator. The observation of sidebands in the frequency spectrum provides direct evidence of parametric coupling between the SAW cavity and cantilever modes. Importantly, the sidebands are observed only when the RF pump tone is tuned at around the SAW cavity resonance, and their amplitudes decrease as the RF pump is detuned further from the cavity mode (see Supplementary Information S1).

The differences in sideband amplitudes between SAW mode’s coupling to bending and torsional modes suggest that the spatial distribution and orientation of strain relative to the SAW propagation direction determine the modulation efficiency. The larger sideband amplitudes in the bending mode can be attributed to the strain in the direction of propagation, which increases the parametric modulation of the SAW cavity frequency. In contrast, torsional modes induce transverse strain. SAW velocity is predominantly affected by strain in the propagation direction, while transverse strain has a negligible contribution^30^. This suggests that the weaker coupling observed for torsional modes in our experiment is consistent with the theoretical expectation that SAWs are more sensitive to in-plane strain components.

The observed modulation can be described as a frequency modulation of the SAW cavity resonance induced by the cantilever motion. The periodic strain generated by the vibrating cantilever modulates the propagation path length of the SAW cavity, thereby modulating its resonance frequency. This frequency modulation produces the carrier power into sidebands at *ω*_*c*_ ± *Ω*_*m*_, and the sideband amplitudes increase with modulation index. In Fig. 4b, the modulation coefficients *a* and *b* from Eq. (3) provide a simple way to quantify how the observed sideband amplitudes vary with cantilever amplitude for different vibrational modes. The first-order modulation coefficient *b* reflects the initial slope of the sideband response at low displacements. Larger *b* values, as in B2 and B3, are consistent with bending modes that generate strain components aligned with the SAW propagation direction.

The second-order modulation coefficient *a* characterizes the curvature of the sideband response with increasing displacement. Large *a* values, as in B1 and T1, indicate that the modulation grows more rapidly at larger amplitudes, suggesting that these mode shapes continue to enhance the relevant strain components with increasing displacement. By contrast, negative *a* values, as in B2, reflect a saturation tendency, where the sideband amplitudes remain large, but their rate of increase diminishes at higher displacements. While the coupling between the SAW cavity mode and the first bending mode (B1) is described by the standard Hamiltonian, it remains an open question whether this model can be applied to higher-order bending modes (B2, B3) and torsional modes (T1, T2). Unlike B1, which follows a clear quadratic dependence in agreement with the calculations, the sideband power trends for other modes do not exhibit a simple quadratic trend, as shown in Fig. 4. The Hamiltonian used in our analysis assumes a linear parametric interaction, which effectively captures the mode coupling for B1, not other modes that might require additional nonlinear interaction terms in the Hamiltonian. Future research could focus on deriving an extended Hamiltonian that supports these nonlinear effects and validates it with additional experimental measurements.

We derive the coupling strength *g*_0_ in our mechano-mechanical system using established principles from cavity optomechanics and compare it with previously studied systems in Table 2. The coupling strength *g*_0_ quantifies the interaction strength in such systems, where a large *g*_0_ enhances performance in applications such as parametric cooling, quantum squeezing, and quantum transduction. Among optomechanical systems, strong coupling has been demonstrated in work by Chan et al.^18^ and Safavi-Naeini et al.^31^, where the high-frequency optical cavity mode (*ω*_*c*_/2*π* ~ 10^14^ Hz) and GHz-range mechanical resonances enable large coupling strengths (*g*_0_ ~ 10^6^ Hz). However, in some cases, minimizing *g*_0_ is necessary to ensure measurement stability, as seen in the work of Biswas et al.^32^, where an extremely small coupling strength (*g*_0_ ~ 10^−29^ Hz) was intentionally designed to optimize gravitational wave detection. Electromechanical systems typically achieve coupling strength *g*_0_ values comparable to those in conventional optomechanical systems. For example, Pirkkalainen et al.^33^ demonstrate coupling strength *g*_0_ ~ 10^7^ Hz using a quantum two-level system (qubit). A useful benchmark is the electromechanical system reported by Scarano et al.^34^, which operates at similar cavity and oscillator frequencies (*ω*_*c*_/2*π* = 4.4 × 10^9^ Hz, *Ω*_*m*_/2*π* = 6.9 × 10^5^ Hz) but reports coupling strength *g*_0_ = 5.0 × 10^−2^ Hz, approximately an order of magnitude larger. However, their measurements were performed at 10 mK in a dilution refrigerator, whereas our system operates at room temperature in air. Magno-mechanical systems, such as those in the works of Amazioug et al.^35^ and Zhang et al.^36^, present an alternative hybrid approach where magnon-phonon interactions are used. These systems typically operate at higher frequencies (*ω*_*c*_ ~ 10^10^ Hz) than our system and exhibit larger coupling strengths in the range of *g*_0_ ~ 10^−2^ − 10^0^ Hz. However, their reliance on magnetostrictive effects fundamentally differentiates them from our purely mechanical implementation. These comparisons establish our system as a relevant cavity-based parametric coupling scheme.

**Table 2:** Comparison of coupling strengths *g*_0_ with different cavity-based parametric coupling systems

System	*ω*_*c*_/2*π* (Hz)	*Ω*_*m*_/2*π* (Hz)	*g*_0_ (Hz)	Reference
Opto-mechanical	2.8 ⋅ 10^14^	3.5 ⋅ 10^9^	7.5 ⋅ 10^6^	^18^
Opto-mechanical	2.0 ⋅ 10^14^	9.3 ⋅ 10^9^	1.4 ⋅ 10^6^	^31^
Opto-mechanical	7.4 ⋅ 10^9^	5.4 ⋅ 10^7^	2.7 ⋅ 10^2^	^39^
Opto-mechanical	7.5 ⋅ 10^9^	1.1 ⋅ 10^7^	1.4 ⋅ 10^2^	^47^
Opto-mechanical	3.0 ⋅ 10^14^	1.5 ⋅ 10^2^	~ 10^−29^	^32^
Electro-mechanical	4.9 ⋅ 10^9^	6.5 ⋅ 10^7^	1.0 ⋅ 10^7^	^33^
Electro-mechanical	4.5 ⋅ 10^9^	5.4 ⋅ 10^6^	6.2 ⋅ 10^1^	^48^
Electro-mechanical	7.0 ⋅ 10^9^	1.9 ⋅ 10^3^	1.3 ⋅ 10^0^	^49^
Electro-mechanical	4.4 ⋅ 10^9^	6.9 ⋅ 10^5^	5.0 ⋅ 10^−2^	^34^
Electro-mechanical	1.0 ⋅ 10^10^	3.2 ⋅ 10^2^	1.3 ⋅ 10^−3^	^50^
Magno-mechanical	1.0 ⋅ 10^10^	1.0 ⋅ 10^7^	1.2 ⋅ 10^0^	^35^
Magno-mechanical	7.9 ⋅ 10^9^	1.1 ⋅ 10^7^	6.2 ⋅ 10^−2^	^36^
Mechano-mechanical	1.2 ⋅ 10^9^	2.7 ⋅ 10^5^	1.1 ⋅ 10^−3^	This work

Furthermore, the coupling strength *g*_0_ can be expressed as ${g}_{0}={x}_{{\text{ZPF}}}\,G=\sqrt{\hslash /(2{m}_{{\rm{e}}\mathrm{ff}}{\Omega }_{m})}\,(\partial {\omega }_{c}/\partial L)$. Increasing the SAW cavity mode frequency *ω*_*c*_ can enhance *g*_0_, since shorter wavelengths make the cavity resonance more sensitive to a given displacement (cavity length). As demonstrated in optomechanical and electromechanical systems, higher cavity frequencies *ω*_*c*_ lead to higher *g*_0_, as shown in Table 2. In our system, one practical way to increase the SAW cavity frequency *ω*_*c*_ is to use advanced nanofabrication techniques such as electron-beam lithography to reduce the electrode pitch down to 200 nm. This would enable operation at a five times higher resonance frequency than that of our current SAW cavity mode. Another approach is to increase the SAW velocity by employing multilayer substrates. For example, the AlN/diamond substrate has an extremely high SAW velocity of about 12,000 ms^−1^^37^. SAW resonators based on this structure which has fine electrodes on heterostructure, have been reported to operate above 10 GHz^38^. According to Eq. (6), another way to enhance the coupling strength *g*_0_ is to increase the zero-point fluctuation amplitude *x*_ZPF_, which is inversely proportional to the square root of the motional mass *m*_eff_. This has been demonstrated by using nanoscale mechanical resonators such as graphene and carbon nanotube (CNT) resonators^39–41^. This strategy could be implemented in our system by replacing the silicon cantilever with a nanoscale resonator, such as a beam-shaped graphene ribbon or CNT suspended over a silicon trench. Two IDTs can be placed on the silicon substrate, generating a SAW cavity on the surface of the nanoscale resonator. While SAWs propagate within a few micrometers below the surface of a piezoelectric substrate and graphene, being only one atomic layer thick, may therefore couple only weakly to the SAW, experimental studies have already demonstrated the feasibility of SAW–graphene coupling^42^. This structure would drastically reduce the effective mass *m*_eff_ while maintaining the SAW cavity.

Although the coupling strength *g*_0_ is a fundamental parameter characterizing the intrinsic interaction strength of the parametric coupling system, it is determined by the effective mass, the resonance frequency, and the geometry parameters of the resonators. Importantly, *g*_0_ is independent of the quality factors and temperature. In cavity optomechanics, the parametric coupling works in the strong-coupling regime *g*_0_ ≫ *κ*, which means that the quality factor of the mechanical modes is a key parameter of the parametric coupling system. Our current system lies in the weak coupling regime *g*_0_ ≪ *κ*, which limits quantum effects but still allows sideband generation and motivates further improvement in quality factors. The SAW cavity shows an internal quality factor *Q*_*i*_ = 4328 under critical coupling conditions, where *Q*_*e*_ ≈ *Q*_*i*_. Under such conditions, the total quality factor *Q* is ~2000, and the cavity decay rate is given by *κ* = *κ*_*e*_ + *κ*_*i*_. In this regime, phonons modulated by mechanical interactions within the cavity are efficiently coupled out through the external port, allowing sidebands to be clearly observed in the reflection spectrum. To access quantum phenomena, such as resolved sideband cooling, phonon squeezing, or quantum backaction, future studies aim to operate the system in the resolved sideband regime (*Ω*_*m*_ ≫ *κ*), which requires enhanced total quality factors. This can be achieved by reducing intrinsic losses through cryogenic operation to increase *Q*_*i*_, while simultaneously improving the Q-factor of the cantilever modes to reduce mechanical dissipation and to increase phonon coherence times. Additionally, the external quality factor *Q*_*e*_ can be tuned using impedance matching networks, as demonstrated in our experiment under critical coupling conditions (*κ*_*e*_ = *κ*_*i*_), which is known to optimize cooling efficiency in cavity-based systems^10,43^.

The ability to observe mechano-mechanical coupling over vast frequency intervals under ambient conditions highlights its potential as a scalable and robust platform for MEMS-based applications. For example, one of the most studied MEMS applications is mass sensing, which requires high mass resolution, down to the attogram level under ambient conditions. To achieve this, MEMS resonators such as cantilevers and drum-like structures have been developed over many years, with efforts focused on reducing thermal noise and improving the signal-to-noise ratio. A strategy involves operating the resonator in higher-order modes to increase its resonance frequency (from kHz toward MHz regime), thereby enhancing frequency stability and sensing resolution^44,45^. In this context, the GHz–kHz mechano-mechanical coupling demonstrated in this study can offer a new sensing strategy. By modulating kHz-frequency modes into GHz-frequency sidebands of the SAW cavity resonance, the system provides a pathway toward detection schemes less affected by thermal noise. The thermal force noise spectral density *S*_*F*_ = 4*k*_*B*_*TmΓ*_*m*_ is frequency independent, but its impact on sensing is governed by the fractional frequency fluctuation *δf*/*f*_0_, which decreases with increasing resonance frequency. Furthermore, because the output can be read out directly in the RF domain using a spectrum analyzer, this approach eliminates the need for laser-based interferometric detection. While our study does not explicitly focus on thermal noise reduction, it represents a crucial first step toward integrating cavity-based parametric coupling into MEMS technologies.

At the same time, the mechano-mechanical coupling we demonstrate offers a new perspective for exploring quantum phenomena traditionally investigated in cavity optomechanics. Approaching the quantum regime requires minimizing the thermal occupation number *n*_th_ ≈ *k*_*B*_*T*/*ℏω*_*c*_, reaching *n*_th_ ≈ 1 at room temperature (*T* = 300 K) would require *ω*_*c*_/2*π* on the order of several terahertz. Although this THz regime is far beyond the current SAW technology, recent advances have pushed SAW resonance frequencies to the 44 GHz^46^. This relation implies that *ω*_*c*_/2*π* must reach approximately 1 GHz for *n*_th_ ≈ 1 at 10 mK in modern cryostats. Our SAW cavity already operates around 1.2 GHz, indicating that further frequency scaling and quality-factor enhancement represent realistic routes to lowering *n*_th_ and approaching quantum operation. Although our experiments are conducted at room temperature, future studies at lower temperatures could reveal quantum effects such as squeezing and ground-state cooling. Moreover, the observation of second-order sidebands indicates that the platform exhibits higher-order parametric interactions beyond the first-order sidebands, suggesting future studies on phenomena such as phonon blockade and effective Kerr nonlinearities. These complementary roles, as a MEMS platform for practical applications and a bridge to quantum mechanics, position the mechano-mechanical system as a promising pathway for advancements in both classical and quantum domains. One relevant application is AFM, where ongoing efforts aim to approach the fundamental limits of force detection. Our approach, where GHz-frequency SAW modes serve as carriers that encode low-frequency cantilever modes, can enable highly sensitive AFM force sensors based on the principles of cavity optomechanics. In this system, the AFM cantilever mode has the potential to modulate the resonance of a GHz SAW cavity, allowing multi-frequency pumping without relying on optical readout. In this sense, our work not only provides a MEMS platform for practical sensing applications but also lays the foundation for connecting conventional MEMS with emerging quantum technologies via mechano-mechanical coupling.

## Methods

### Device fabrication process

The device used in this study is fabricated on a silicon-on-insulator (SOI) wafer with a 10 μm-thick device layer, a 1 μm-thick buried oxide (BOX) layer, and a 400 μm-thick handle layer. An 80 nm silicon dioxide (SiO_2_) and 240 nm silicon nitride (Si_3_N_4_) layer are deposited on both the front and back sides of the wafer. A piezoelectric AlN thin film with a thickness of 1 μm is sputter-deposited on the SOI wafer using a DC magnetron sputtering system (LS730S, Von Ardenne) at a chamber pressure of 2 μbar in a pure nitrogen atmosphere, with a constant flow rate of 50 sccm and plasma power of 800 W. The fabrication process involves four photolithography steps. First, the IDTs and reflectors are patterned on the AlN layer using an AZ5214 photoresist, followed by metal deposition of a Cr/Al/Au layer (50 nm/250 nm/50 nm) via PVD evaporation. The structures are formated by a lift-off process in acetone. Second, the cantilever outline is defined by photolithography, and the AlN layer is etched using a Scia dry etching system. Third, lithography is performed on the front side to define the cantilever geometry, followed by deep reactive ion etching (DRIE). Fourth, the back side of the wafer undergoes lithography and DRIE etching, after which the handle layer is released using buffered oxide etching (HF, 20 min) and cleaned with deionized (DI) water. The resulting devices integrate a GHz SAW cavity mode and a cantilever structure with precise dimensions, ready for experimental characterization.

### Measurement setup

The measurement setup in this study is designed to first characterize the individual modes of the cantilever and the SAW resonator separately, followed by studying their interaction when coupled. For the characterization of the cantilever’s mechanical modes, we use an LDV system (MSA 500, Polytec) to scan the cantilever surface and measure its displacement across a range of frequencies. The cantilever is driven by a PZT actuator attached beneath the silicon cantilever chip, allowing us to identify its resonance frequencies and mode shapes. In this step, data on mechanical bending and torsion modes are obtained. During this process, only the cantilever mode is analyzed, and the SAW mode is not actively excited. Next, the SAW cavity mode is characterized independently using a VNA (Rohde & Schwarz ZVL-6) and an impedance tuner. The impedance tuner optimizes power transfer to the SAW cavity, and the VNA monitors the interaction. The GHz SAW mode is generated by the sputter-deposited AlN thin film integrated into the device. The VNA measures the reflection coefficient (*S*_11_) to determine the resonance frequency and quality factor of the SAW cavity. At this stage, the cantilever is not vibrated, focusing entirely on the SAW resonator.

To study the coupling between the cantilever and SAW modes, the two systems are combined. Fig. 1e. illustrates the complete measurement setup used to study the coupled system. The combined setup includes both the LDV system for cantilever actuation and mode characterization and the electrical components for SAW excitation and detection. The cantilever is driven at its resonance frequency determined during the initial characterization, using the PZT actuator. Simultaneously, the SAW cavity mode is excited by applying a 10 dBm signal at 1.20878 GHz to the AlN thin film using the signal generator. The signal then passes through a −6 dB coupler, which attenuates the power before reaching the device. The coupler also separates the incident and reflected signals, allowing the VNA to accurately measure the SAW resonance characteristics while ensuring efficient power delivery to the device. The VNA switches to a spectrum analyzer mode, and this configuration enables the observation of sidebands in the frequency spectrum.

## Supplementary information

Supplementary information for: Mechano-mechanical parametric coupling in MEMS between GHz and kHz frequency regimes at room temperature
