# Supplementary material for: Mechano-mechanical parametric coupling in MEMS between GHz and kHz frequency regimes at room temperature
Source: Microsyst Nanoeng. 2026 Jan 9;12:24. doi: 10.1038/s41378-025-01111-1 (PMC12783657; doi:10.1038/s41378-025-01111-1)
Supplement: Supplementary file 1 — Supplementary information for: Mechano-mechanical parametric coupling in MEMS between GHz and kHz frequency regimes at room temperature [file 41378_2025_1111_MOESM1_ESM.pdf]

Supplementary information for:

Mechano-mechanical parametric coupling in MEMS between GHz and kHz frequency regimes at room temperature

MinHee Kwon[1,*], Holger Arthaber[2], Daniel Platz[1], Ulrich Schmid[1]

[1]Institute of Sensor and Actuator Systems, TU Wien, Gusshausstrasse 27-29, 1040 Vienna, Austria.
[2]Institute of Electrodynamics, Microwave and Circuit Engineering, TU Wien, Gusshausstrasse 27-29, 1040 Vienna, Austria.

[*]Corresponding Author: minhee.kwon@tuwien.ac.at

S1. Dependence of sideband generation on RF pump frequency

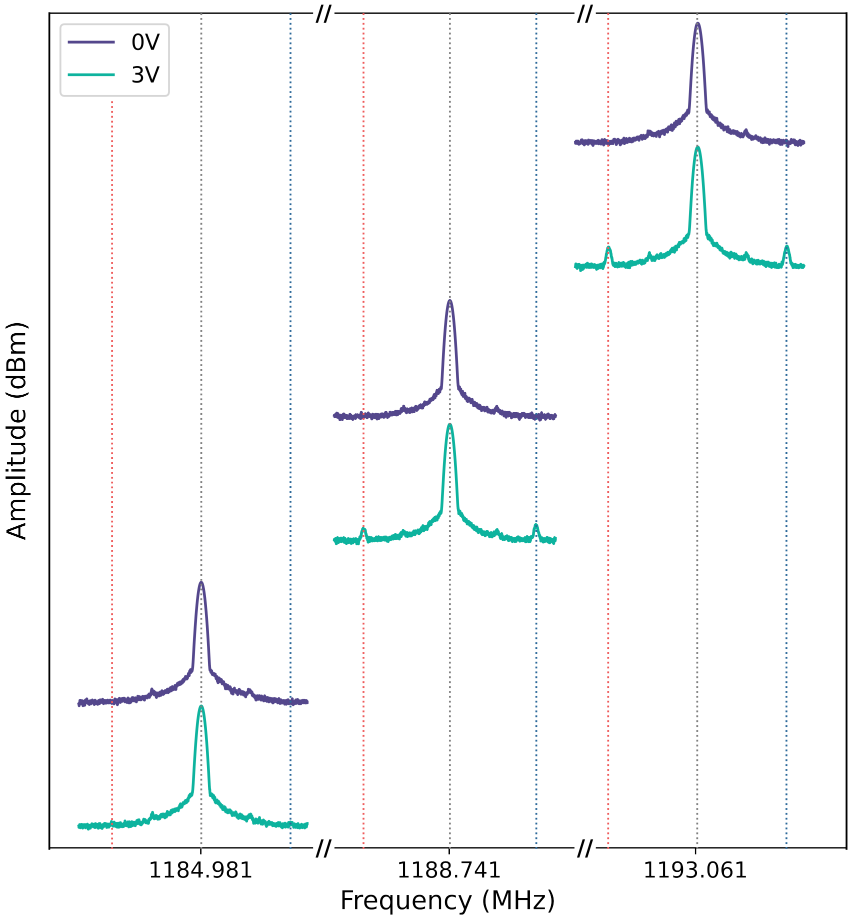

Fig. S1 Observation of sidebands under different RF pump frequencies. Measured frequency spectra are shown for three different RF pump frequencies: 1193.061 MHz (resonance frequency with SAW cavity mode), 1188.741 MHz, and 1184.981 MHz. The cantilever is driven at its first bending mode (6.82 kHz) using a PZT actuator with 0 V (purple) and 3 V in all cases. The gray dotted lines indicate the RF pump tone, while the red and blue dotted lines indicate the positions at the frequency of the cantilever's first bending mode away from each pump tone. Each data set is vertically offset for visual comparison.

To observe that sideband generation appears only when the RF pump tone is matched to the SAW cavity resonance frequency, another mechano-mechanical device is performed in the same

setup as the one presented in this paper. The device consists of 50 pairs of IDT electrodes with a pitch size of 1 µm and 250 reflector electrodes, and the IDT aperture width is 280 µm. The length of the cantilever 1000 µm, which is twice as long as the length of the cantilever in this paper, and the first bending mode is 6.82 kHz. The RF pump was operated at three frequencies, 1193.061 MHz, which match the SAW cavity resonance, 1188.741 MHz, and 1184.981 MHz. At each RF tone frequency, the cantilever was vibrated at 0 and 3 V using a PZT actuator. The resulting spectra are shown in Figure S1. When the RF pump is matched to the SAW cavity resonance (1193.061 MHz), the red and blue sidebands are symmetrically separated by the bending mode 6.82 kHz. As the RF pump is detuned from the SAW mode, the sideband amplitudes are decreased at 1188.741 MHz. At 1184.981 MHz sidebands are not observed. This shows that the observed sidebands arise from the parametric coupling between the GHz SAW cavity mode and the kHz cantilever mode.

S2. Sidebands from higher-order cantilever modes

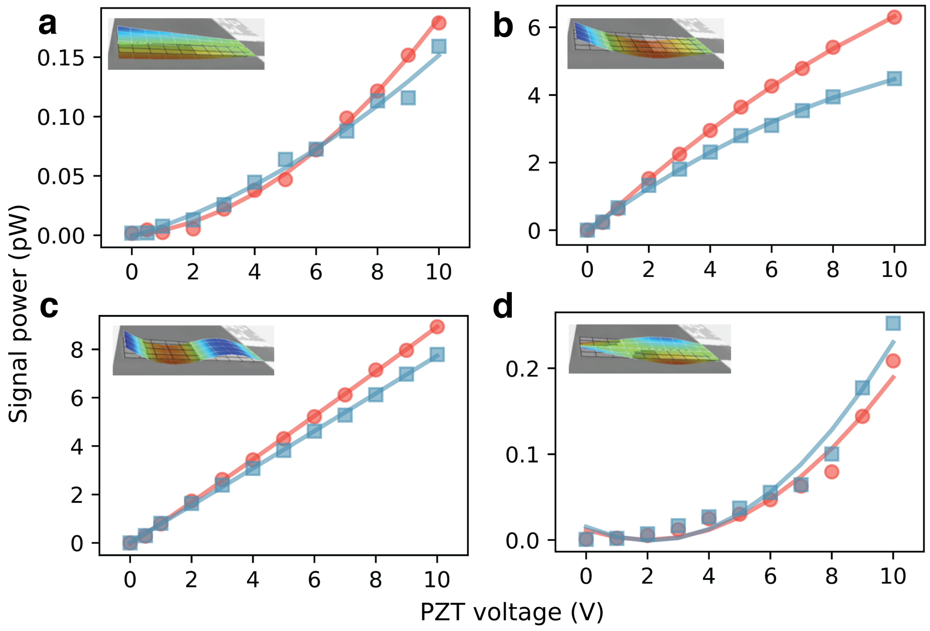

Fig. S2 Measured sidebands for higher-order cantilever modes. The red and blue sidebands are plotted for each mode: **a** T1, **b** B2, **c** B3, **d** T2. The red circles and blue squares represent the measured red and blue sideband power, respectively, as a function of PZT voltage. The solid lines are quadratic fits. Insets show the corresponding mode shapes obtained from LDV measurements.

S3. Calculation of the effective mass $m_{\mathbf{eff}}$

The effective mass m_{eff} of the cantilever is determined based on the mode shape function $w(x)$ using Euler-Bernoulli beam theory. For a cantilever beam of length L, the effective mass is given by

$$m_{\text{eff}} = \frac{M}{L}\int_0^L w^2(x)dx, \tag{1}$$

where M is the total mass of the cantilever, and $w(x)$ is the normalized mode shape function. The mode shape function for the nth bending mode is expressed as

$$w_n(x) = (\cosh\beta_n x - \cos\beta_n x) + \frac{\cos\beta_n L + \cosh\beta_n L}{\sin\beta_n L + \sinh\beta_n L}\left(\sin\beta_n x - \sinh\beta_n x\right), \tag{2}$$

where β_n is the characteristic eigenvalue for each mode. These eigenvalues satisfy the boundary condition for a beam with one end fixed, given by

$$\cosh(\beta_n L)\cos(\beta_n L) + 1 = 0. \tag{3}$$

The first three solutions of this equation are $\beta_1 L = 0.596864\pi$, $\beta_2 L = 1.49418\pi$, and $\beta_3 L = 2.50025\pi$. The mode shape function normalized is defined as

$$w_n^{\text{norm}}(x) = \frac{w_n(x)}{w_n(L)}, \tag{4}$$

where $w_n(L)$ is the maximum displacement of the cantilever. The total mass of the cantilever is given by

$$M = \rho A L, \tag{5}$$

where ρ is the density of silicon, A is the cross-sectional area, and L is the total length of the cantilever. The values used in this study are $\rho = 2330$ kg/m^3, $A = 308$ μm $\times$ 11 μm, and $L = 1000$ μm. Since the mode shape function is normalized, the effective mass is computed as Eq. (1). The calculated effective masses and their ratios relative to the total mass are presented in Table 1.

Table 1 Effective mass m_{eff} and its ratio to the total cantilever mass M for the first three bending modes.

Mode	Effective Mass m_{eff} (kg)	Ratio m_{eff}/M
B1	1.115×10^{-9}	0.141
B2	8.400×10^{-10}	0.106
B3	5.025×10^{-10}	0.064

S4. Calculation of the cavity length change ΔL

The cavity length change ΔL is determined by the strain $\epsilon(x)$ along the cantilever length L. The strain $\epsilon(x)$ is given by

$$\epsilon(x) = -z \cdot \frac{d^2 w(x)}{dx^2}, \tag{6}$$

where $z = t/2$ is the distance from the neutral axis, and $w(x)$ is the mode shape function. Since the mode shape function is a relative displacement function, its absolute value must be scaled according to the maximum displacement of the cantilever, w_C. The scaled mode shape function is then used to compute the second derivative numerically, and the total cavity length change ΔL is obtained by integrating the strain:

$$\Delta L = \int_0^L \epsilon(x) dx. \tag{7}$$

For the first bending mode (B1), the mode shape function is given by

$$w_1(x) = (\cosh \beta_1 x - \cos \beta_1 x) + \frac{\cos \beta_1 L + \cosh \beta_1 L}{\sin \beta_1 L + \sinh \beta_1 L} (\sin \beta_1 x - \sinh \beta_1 x), \tag{8}$$

where $\beta_1 L = 0.596864\pi$. The computed values for B1, B2, and B3 modes are summarized in Table 2. These values of ΔL are used in Section 2.3 to compute the coupling strength g_0.

Table 2 Calculated cavity length change ΔL for different cantilever modes. The maximum displacement of the cantilever tip, w_C, is used to scale the mode shape function.

Mode	Max. cantilever displacement w_C (m)	Cavity length change ΔL (m)
B1	2.04×10^{-12}	1.58×10^{-11}
B2	3.40×10^{-12}	8.96×10^{-11}
B3	5.85×10^{-12}	2.52×10^{-10}
